# Chronic Administration of Fipronil Heterogeneously Alters the Neurochemistry of Monoaminergic Systems in the Rat Brain

**DOI:** 10.3390/ijms21165711

**Published:** 2020-08-09

**Authors:** Rahul Bharatiya, Abdeslam Chagraoui, Salomé De Deurwaerdere, Antonio Argiolas, Maria Rosaria Melis, Fabrizio Sanna, Philippe De Deurwaerdere

**Affiliations:** 1 Department of Biomedical Sciences, Section of Neuroscience and Clinical Pharmacology, University of Cagliari, 09100 Cagliari, Italy; bhartiyarahul20@gmail.com (R.B.); argiolas@unica.it (A.A.); mrmelis@unica.it (M.R.M.); 2 Centre National de la Recherche Scientifique (Unité Mixte de Recherche 5287), 146 rue Léo Saignat, B.P.281, F-33000 Bordeaux CEDEX, France; salome.de-deurwaerdere@etu.u-bordeaux.fr; 3 Neuronal and Neuroendocrine Differentiation and Communication Laboratory, Institute for Research and Innovation in Biomedicine of Normandy (IRIB), Normandie Univ, UNIROUEN, INSERM, U1239, CHU Rouen, 76000 Rouen, France; abdeslam.chagraoui@univ-rouen.fr; 4 Department of Medical Biochemistry, Rouen University Hospital, 76000 Rouen, France; 5 Centre of Excellence for the Neurobiology of Addictions, University of Cagliari, 09100 Cagliari, Italy; 6 Institute of Neuroscience, National Research Council, Cagliari Section, University of Cagliari, 09100 Cagliari, Italy

**Keywords:** pesticide, dopamine, serotonin, nigrostriatal, metabolism, high pressure liquid chromatography

## Abstract

Fipronil (FPN), a widely used pesticide for agricultural and non-agricultural pest control, is possibly neurotoxic for mammals. Brain monoaminergic systems, involved in virtually all brain functions, have been shown to be sensitive to numerous pesticides. Here, we addressed the hypothesis that chronic exposure to FPN could modify brain monoamine neurochemistry. FPN (10 mg/kg) was chronically administered for 21 days through oral gavage in rats. Thereafter, the tissue concentrations of dopamine (DA) and its metabolites, 3,4-dihydroxyphenylacetic acid (DOPAC) and homovanillic acid; serotonin (5-HT) and its metabolite, 5-hydroxyindoleacetic acid (5-HIAA); and noradrenaline (NA) were measured in 30 distinct brain regions. FPN significantly decreased DA and its metabolite levels in most striatal territories, including the nucleus accumbens and the substantia nigra (SN). FPN also diminished 5-HT levels in some striatal regions and the SN. The indirect index of the turnovers, DOPAC/DA and 5-HIAA/5-HT ratios, was increased in numerous brain regions. FPN reduced the NA content only in the nucleus accumbens core. Using the Bravais–Pearson test to study the neurochemical organization of monoamines through multiple correlative analyses across the brain, we found fewer correlations for NA, DOPAC/DA, and 5-HIAA/5-HT ratios, and an altered pattern of correlations within and between monoamine systems. We therefore conclude that the chronic administration of FPN in rats induces massive and inhomogeneous changes in the DA and 5-HT systems in the brain.

## 1. Introduction

Pesticides are widely used in agricultural and non-agricultural pest control, but their use is a matter of public health [1,2]. Past pesticides, such as rotenone, have been associated with the development of neurological conditions, including Parkinson’s disease and Alzheimer’s disease [1,3,4]. Other toxic strategies toward insects have been developed, leading to new generations of compounds. Fipronil (FPN), a type class-C of pesticides, is one of these drugs which has been extensively used [5]. However, FPN is suspected to produce noxious effects in various tissues, including the brain [5].

The earlier reports suggest that FPN blocks chloride ion cellular uptake in invertebrates, leading to uncontrolled central nervous system (CNS) hyper-excitation by the blockage of GABA_A_ receptors, convulsion, and cell death [6,7]. Although considered less toxic toward mammals, its absorption is rapid, and it easily crosses the blood brain barrier [8]. Recent alarming reports suggest that chronic FPN in rodents at 30 but not 10 mg/kg by daily oral gavage causes memory impairment in parts associated with the modulation of the GABAergic system [9] and the deposition of amyloid plaques [10]. FPN oral administration raises the oxidative stress in various tissues, including the adipose tissue, the liver, and the brain, in a range of doses of 5–30 mg/kg [8,11,12,13]. Upon intra-nigral administration in rats, FPN reduced the dopamine (DA) content in the striatum as well as the tyrosine hydroxylase (TH) levels in the striatum and the substantia nigra (SN) pars compacta, and impaired motor coordination [14,15]. It remains to be established whether the toxic effects of FPN occur upon its oral absorption into the nigrostriatal DAergic system, and, by extension, into the other monoaminergic systems.

The monoaminergic systems serotonin (5-HT), DA, and noradrenaline (NA) exert complex neuromodulation on motor, cognitive, affective, and neuroendocrine functions, and a noxious action of FPN on monoaminergic systems could predispose individuals to develop neuropsychiatric diseases. It is possible to address the effect of FPN on monoamine systems by measuring the content of the monoamines and their metabolites in various parts of the brain. It allows one to determine possible regional and quantitative alterations in the neurochemistry of monoamines at terminals in response to neurotoxins, including 1-methyl-4-phenyl-pyridinium ion (MPTP) [16,17], rotenone [18], 6-hydroxydopamine (6-OHDA) [19], or paraquat and maneb [20], to cite a few. However, it is noticeable that numerous studies have failed to report an effect of the full lesion of one monoaminergic system on the tissue content of the other monoamines [21,22], at odds with the numerous local and distal interactions these systems establish [23,24,25,26,27]. Thus, beyond quantitative analysis, it is possible to qualitatively address the neurochemical re-organization of monoamine systems across the brain and study their metabolism [28] using multiple linear regressions of monoamine content between pairs of brain regions [29,30]. Considering the previous studies showing that FPN impairs oxidative metabolism and motor and cognitive functions (see above), it is hypothesized that FPN, upon its oral administration, could disrupt the functional and biochemical organization of monoamines in the brain, possibly modelling some features of the pathophysiology of Parkinson’s disease.

**Figure 1:**
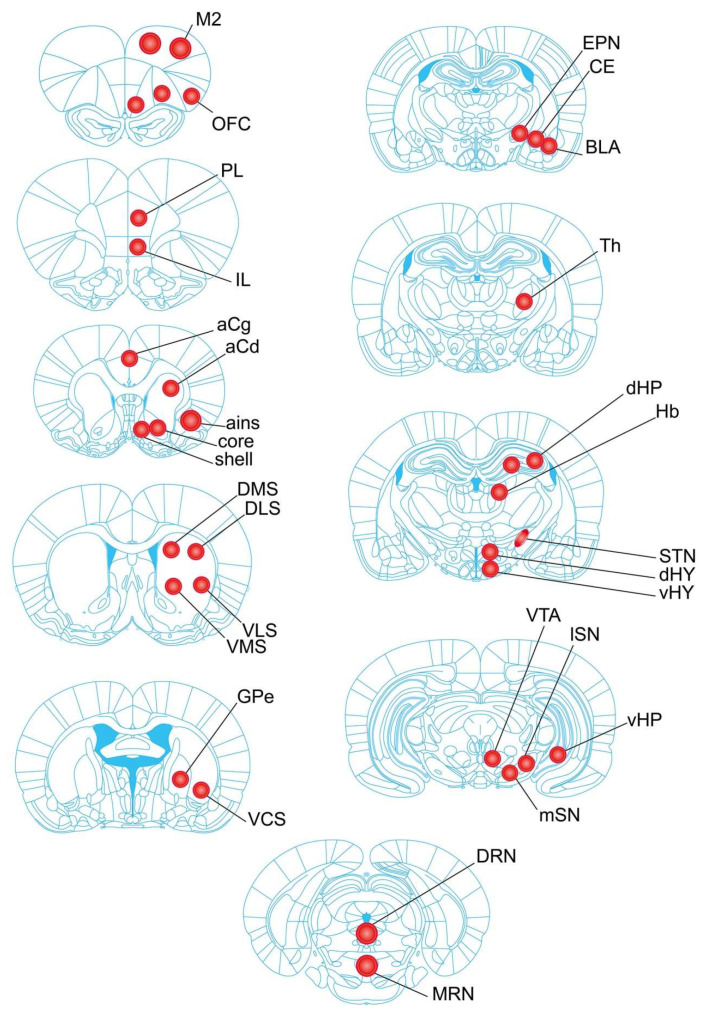
The pictures illustrate the approximate location of the punched brain region (adapted from Paxinos and Watson, 2007). Tissue samples were taken from left and right cerebral hemispheres (mean size in mg ± SEM) in a cryostat using a punch of 500 µm (smaller circles in pictures) or 800 µm (largest circles) inner diameter, except for the subthalamic nucleus (STN). Cortical areas: orbitofrontal cortex (OFC, 2.04 ± 0.13), motor cortex M2 (3.09 ± 0.11), prelimbic (PL, 1.62 ± 0.08) and infralimbic (IL, 2.08 ± 0.11) cortices, anterior cingulate cortex (aCg, 1.86 ± 0.15), anterior insular cortex (ains, 1.95 ± 0.09). Sub cortical areas: nucleus accumbens—shell (1.90 ± 0.12) and core (1.77 ± 0.10); striatum—anterior caudate (aCd, 2.63 ± 0.16), dorsomedial (DMS, 2.09 ± 0.09), dorsolateral (DLS, 2.03 ± 0.09), ventromedial (VMS, 2.03 ± 0.13), ventrolateral (VLS, 2.17 ± 0.14), and ventrocaudal (VCS, 2.33 ± 0.15) striatum; globus pallidus pars externa (GPe, 1.46 ± 0.07), entopeduncular nucleus (EPN, 1.45 ± 0.07), dorsal and ventral hippocampus (dHP, 2.14 ± 0.08 and vHP, 1.98 ± 0.11), habenula (Hb, 1.81 ± 0.09), Thalamus (Th, 2.17 ± 0.07), STN (2.35 ± 0.15); amygdala (basolateral nucleus (BLA, 1.37 ± 0.05) and central nucleus (CE, 1.37 ± 0.06)); dorsal and ventral parts of the hypothalamus (dHY, 1.05 ± 0.07 and vHY, 1.33 ± 0.06); substantia nigra—medial part (mSN, 1.71 ± 0.07) and lateral part (lSN, 1.60 ± 0.09); ventral tegmental area (VTA, 1.56 ± 0.10); dorsal raphe nucleus (DRN, 1.60 ± 0.06) and median raphe nucleus (MRN, 1.79 ± 0.07). Two punched tissues in each side from the M2, and three punched tissues from the OFC and the dHP regions were taken in order to measure the concentrations of monoamines (particularly DA).

The aim of this study was to determine the potential noxious effects of chronic exposure to FPN on monoamine systems in various regions of the rat brain. Among the doses that have been previously found to be noxious in rodents (5–30 mg/kg, [9,10]), we have selected the dose of 10 mg/kg FPN, which was given chronically once a day for a period of 21 days in rats by oral gavage. The monoamines were measured using HPLC-electrochemical detection (ECD) in 30 distinct parts of the rat brain from the cortical areas to the mesencephalic nuclei, with specific attention to the striatal sub-regions (Figure 1). These brain regions were selected because they receive various degrees of monoaminergic innervation from the ventral tegmental area (VTA) and the substantia nigra (SN) for DA [31,32], the median and dorsal raphe nuclei (MRN and DRN) for 5-HT [33,34], and the locus coeruleus for NA [35]. Furthermore, the selected brain regions belong to distinct neurobiological networks involved in cognition, motor behavior, mood, and vegetative functions, which allow for addressing possible monoaminergic imbalances across the brain.

## 2. Results

### 2.1. Body Weight and Behavior

**Figure 2:**
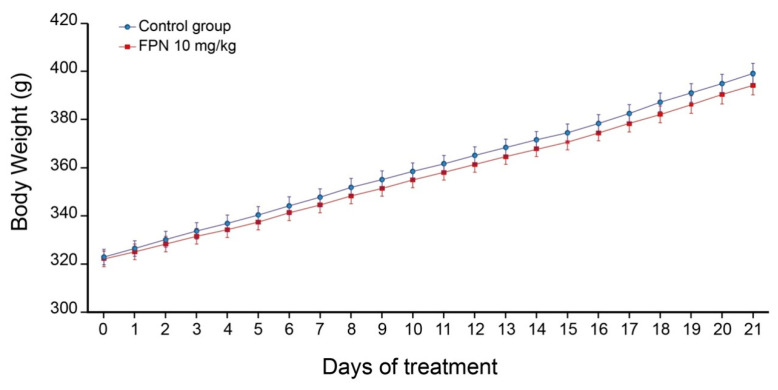
Changes in the body weight gain of the control water-treated rats and FPN (10 mg/kg)-treated rats. Values are expressed as mean ± SEM (*n* = 12 rats per group).

The changes in body weight gain in the control rats and those exposed to chronic treatment with FPN (10 mg/kg) for 21 days are given in Figure 2. The results showed that there is no significant difference in the body weight gain pattern of the FPN-treated and the control water-treated rats (repeated measures ANOVA, F(21, 462) = 1.3, not significant]. A slight, though non-significant, decrease in body weight was observed in the FPN-treated rats. No evident signs of motor impairment or aggressive behavior were noticed, but we did not score any of these behaviors.

### 2.2. Quantitative Analysis of Monoamine Tissue Contents

We studied the effect of chronic treatment with FPN (10 mg/kg/day, oral gavage) for 21 days on the quantitative distribution of NA, DA, and 5-HT neurochemical indices in 30 brain regions of rats and compared it with the control (water-treated) rats. The quantitative analysis of the effects of FPN on monoamines is reported in Table 1a,b.

#### 2.2.1. Quantitative Analysis of DA System

The tissue levels of DA were largely heterogeneous across the brain regions of the rats (Table 1a). Briefly, the DA levels were very high along the nigrostriatal and mesolimbic areas, reaching up to 5039 ± 435 pg/mg of tissue in the aCd. They were almost equivalent in the striatum (DMS, DLS, and VMS, except in VLS), reaching over 2800 pg/mg of tissue. The DA levels were also elevated in the NAc shell and core, and were lower in the amygdala, STN, SN, and VTA. Conversely, they were very low in the hippocampus (dHP and vHP, with 2.33 ± 0.50 pg/mg and 3.82 ± 1.49 pg/mg of tissue, respectively) and low in the thalamus (8.39 ± 1.28 pg/mg of tissue).

The chronic treatment with FPN significantly decreased the DA levels in all the regions of the striatum (DMS, DLS, VMS, VLS, aCd, and VCS) by 30% to 60% with respect to the control rats. The alteration was thus heterogeneous in the striatum. Lower levels of DA were also reported in both the medial and lateral SN (about 50%), in the motor cortex M2 (about 50%), and in the NAc core (about 27%). Conversely, the DA level was elevated in the PL by approximately +75% in the FPN-treated rats. Moreover, the FPN did not significantly affect the DA levels in other brain regions, including the cortex (OFC, IL, aCg, ains), GPe, amygdala, hypothalamus, hippocampus, VTA, DRN, and MRN.

The distribution of the two main metabolites of DA, the DOPAC and HVA, was similar to the parent neurotransmitter, marked by very high concentrations in the striatum quadrants and the NAc core and shell, moderately high concentrations in the SN or VTA, and poorly present concentrations in the hippocampus (dHP and vHP) (Table 1a). In the FPN-treated rats, the DOPAC and HVA levels were significantly decreased in all the striatal regions, except the VLS, when compared to the control rats. The levels were also significantly decreased in the medial part of substantia nigra (mSN), but not in its lateral part (lSN). The levels of DOPAC, but not HVA, were substantially decreased in the motor cortex M2 of FPN-treated rats. On the other hand, the levels of DOPAC were higher in the hippocampus (dHP and vHP) after chronic FPN treatment, and the levels of HVA were higher only in the vHP. In other brain regions, the levels of DOPAC and HVA were not significantly different between the water- and FPN-treated rats.

The chronic treatment with FPN affected the DA turnover (DOPAC/DA ratio) in a few brain regions. The DOPAC/DA ratio was significantly decreased in the PL of FPN-treated rats in line with the significant elevation of DA levels. Conversely, the DOPAC/DA ratio was substantially increased in DLS as compared to the control rats. The FPN treatment also enhanced the DA turnover in the VLS, VCS, and lSN. FPN treatment increased the DOPAC/DA ratio in VTA and MRN without significantly modifying the DOPAC or DA levels in these areas (Table 1a).

#### 2.2.2. Quantitative Analysis of 5-HT System

Unlike DA, the tissue levels of 5-HT were less heterogeneous across all 30 brain regions. The 5-HT levels were high in DRN (1150 ± 151 pg/mg), MRN (1183 ± 101 pg/mg), SN (845.4 ± 46.6 pg/mg in mSN and 500.1 ± 54.6 pg/mg in lSN), and the VTA (688.2 ± 60.8 pg/mg). The levels of 5-HT were lower in other brain regions, though easily detectable (Table 1b). Chronic FPN treatment induced significant effects on the 5-HT levels in the NAc and striato-nigral regions. The levels in the FPN-treated rats were significantly decreased in the NAc shell and core (about 34% and 29%, respectively), the aCd (about 27%), the VLS and VMS (about 40%), and the VCS (about 38%), but not in the two dorsal parts of the striatum of the FPN-treated rats. As for the DA levels, FPN significantly reduced the 5-HT levels in the medial (about 40%) but not in the lateral SN. FPN did not significantly alter the 5-HT content in the other brain regions, despite some trend toward a decrease (M2, IL, EPN, CE, BLA, STN, and VTA) or an increase (GPe, lSN, vHP, DRN, and MRN) (Table 1b).

The chronic treatment with FPN also modified the pattern of the 5-HIAA levels in a few brain regions (Table 1b). The tissue levels of 5-HIAA in the FPN-treated rats were significantly reduced in the EPN. Like 5-HT, the 5-HIAA levels were decreased in the medial SN with respect to the control rats. It was noteworthy that the 5-HIAA levels were significantly increased in the aCg and DMS after the FPN treatment.

The indirect index of the 5-HT turnover (5-HIAA/5-HT ratio) was markedly increased in a few brain regions after the FPN treatment, including the NAc shell and all quadrants of the striatum. The 5-HT turnover was also substantially increased in the IL, DRN, and MRN of FPN-treated rats without significant changes in the 5-HT or 5-HIAA tissue levels (Table 1b).

#### 2.2.3. Quantitative Analysis of NA Tissue Contents

We also studied the effect of FPN on the tissue levels of NA in rat brain (reported in Table 1b). The highest levels were observed in the hypothalamus (dHY, vHY), VTA, DRN, and MRN. The levels were lower in the other brain regions. They were not detected in some parts of the cortex (including M2 and OFC), the striatum (DMS, DLS, VMS, VLS, aCd), as well as the GPe. Even if the levels are reputedly low in these brain regions, the elution time of NA, usually close to the solvent front [36], was not sufficiently spaced in one of our chromatographic conditions, impairing a good determination of the electrochemical signal, corresponding to NA in these brain regions. Some examples of chromatograms obtained in our conditions are reported in Supplementary Figure S1.

As compared to the control group, FPN significantly decreased the NA level in the NAc core only (about 33%). The NA levels were slightly, though not significantly, decreased by FPN in other regions, such as the EPN, BLA, hypothalamus (dHY, vHY), and medial SN (Table 1b).

### 2.3. Qualitative and Correlative Analysis of Monoamine Tissue Contents

**Figure 3:**
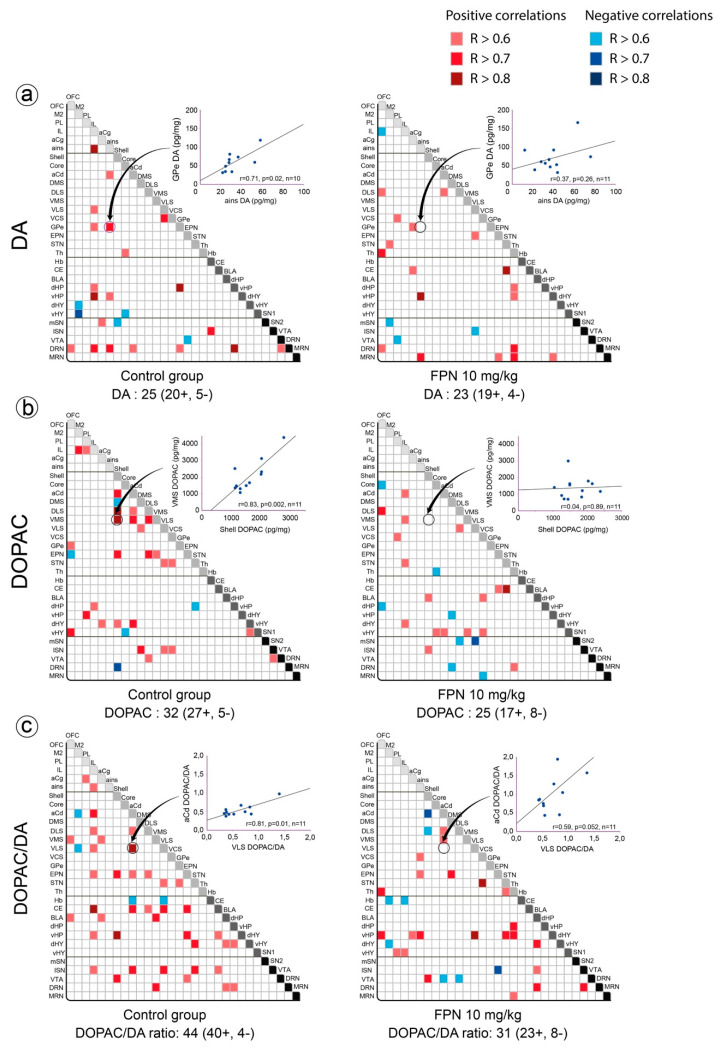
Correlative analysis of the DA content across rat brain regions. Representation of the range of Pearson’s R values for each linear regression of the DA (**a**) and DOPAC (**b**) tissue contents (pg/mg) and the DOPAC/DA ratio (**c**) between the 30 brain areas in the control water-treated rats (first column) and the FPN 10 mg/kg-treated rats (second column). Colored boxes correspond to the existence of a correlation between the two parameters (red: positive; blue: negative), considered after correction for multiple comparisons. The insets for each matrix of correlations correspond to examples of scatter plots reporting the regression lines obtained for series of data.

We have considered the correlations within a single monoaminergic system or between two monoaminergic systems across the numerous investigated brain regions. With the exception of NA (see below), the number of correlations per pair of brain regions across the 30 analyzed brain regions correspond to 420 for a single parameter and 900 for the relationships between parameters. We have arbitrarily reported the number of correlations in the control water-treated versus FPN-treated rats as an indirect index of the possible changes of relationships of monoamines across the brain [37]. We have then reported the changes in the pattern of correlations. Examples of scatter plots reporting the regression lines obtained for series of data are reported in Figure 3. They depict the correlations for the DA tissue content in the ains and GPe (upper panel), the DOPAC tissue content in the shell and VMS, and the ratio of DOPAC/DA in the aCd and VLS in both groups.

#### 2.3.1. Within Monoaminergic Systems

##### Correlative Analysis of DA System

The number of correlations for the DA content in the control group rats was 25, including positive (20) and negative (5) correlations. The DA content in the IL and DRN mostly correlated with that in other brain regions (5 and 6, respectively), whereas the DA content in the OFC, PL, DLS, VMS, Hb, and MRN did not correlate with the other brain regions. FPN did not alter the number of correlations for the DA content (23, comprising 19 positive and 4 negative correlations), but modified the pattern. The DA content of the OFC, Th, and MRN (4–6) established correlations with other brain regions, whereas no correlations were observed for the DA content of the NAc shell and core, the DMS, the BLA, the two parts of the hypothalamus, and the Hb (Figure 3a).

Likewise, the DOPAC content correlated in 32 brain areas (comprising 27 positive and 5 negative correlations) in the control rats, in particular with the NAc shell. The number of correlations slightly decreased (25 correlations) after the FPN treatment in terms of the general pattern; the numerous correlations (13) for DOPAC inside the basal ganglia of the control group were reduced to only one in the FPN-treated rats (Figure 3b).

The HVA content correlated in 25 brain areas, and was mostly positive (only 1 negative correlation) in the control group rats. The number of correlations almost remained similar in the FPN-treated rats (26, 18 positive and 8 negative correlations) (data not shown). The correlations of the HVA content present in the VLS, dHP, vHY, and CE were completely lost after the FPN treatment. On the other hand, the FPN treatment enhanced the correlations of HVA content in the PL with six other brain regions (data not shown).

In line with previous data [29], we observed a higher number of correlations for the DOPAC/DA ratio compared to DA or DOPAC alone (Figure 3c). Regions such as IL, aCd, EPN, and CE established a greater number of correlations with other brain regions (6–8). The chronic treatment with FPN decreased the number of correlations for the DA turnover (31 correlations instead of 44 in water-treated rats), particularly between the cortical and the basal ganglia, the striatum and the amygdala (CE, BLA), and the EPN and the frontal cortex.

##### Correlative Analysis of the 5-HT System

**Figure 4:**
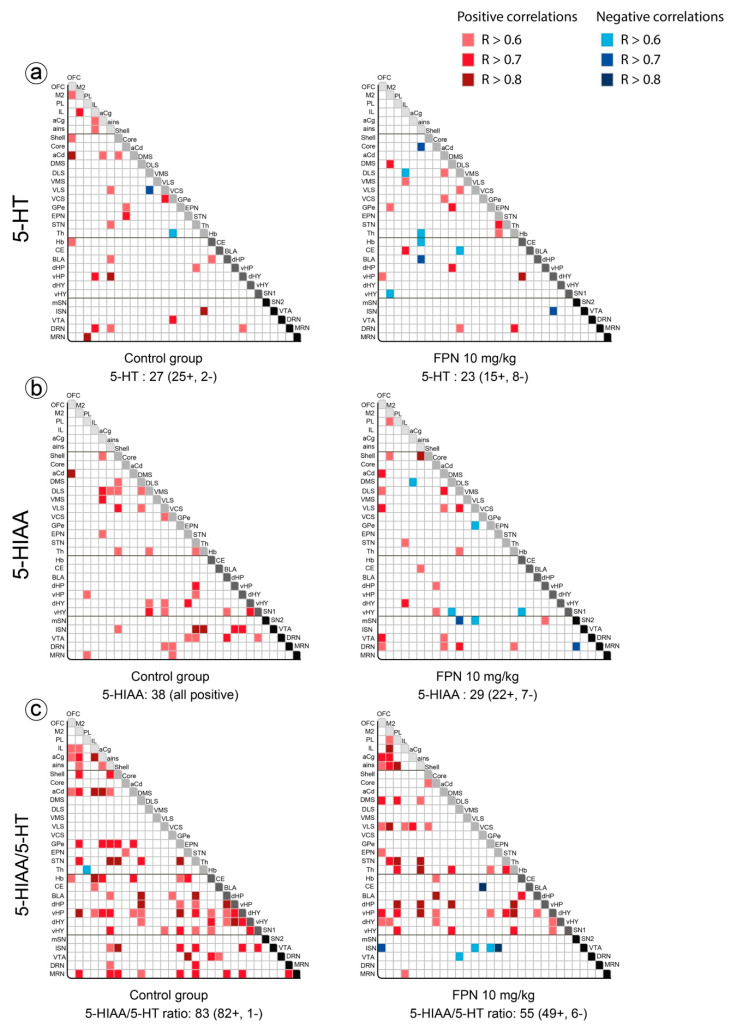
Correlative analysis of the 5-HT content across rat brain regions. Representation of the range of Pearson’s R values for each linear regression of the 5-HT (**a**) and 5-HIAA (**b**) tissue contents (pg/mg) as well as the 5-HIAA/5-HT ratio (**c**) between the 30 brain areas in the control water-treated rats (first column) and the FPN 10 mg/kg-treated rats (second column). Colored boxes correspond to the existence of a correlation between the two parameters (red: positive; blue: negative) considered after correction for multiple comparisons.

The number of correlations for the 5-HT content was slightly reduced in the FPN group compared to the control group (23 and 27 correlations, respectively; Figure 4a). The correlations of the 5-HT content, more prominent in the cortical regions (M2, OFC, IL, ains) and the NAc of the control group, were decreased in the FPN-treated rats. In contrast, the correlations were enhanced in the dorsal striatum (DMS and DLS) and the NAc core with other brain regions.

We observed a higher number of correlations for the 5-HIAA content in the control group (38) compared to the FPN group (29). The correlations in the NAc shell, DLS, VLS, and STN with other brain regions (6–7) of the control rats were reduced in the FPN-treated rats. Likewise, the correlations of the 5-HIAA content in the SN, EPN, and vHP were lost after the FPN treatment. The correlations were increased with the M2, OFC, NAc core, and DRN in the FPN-treated rats (Figure 4b).

The number of correlations for the 5-HIAA/5-HT ratio was higher than 5-HT or 5-HIAA (Figure 4c). The number of correlations was very high with the GPe, STN, and vHP (12–14), and still high with M2, aCg, NAc shell, BLA, Hb, and MRN (8–10). The FPN treatment decreased the number of correlations for the 5-HT turnover too (55 instead of 83 in the control group). The FPN treatment enhanced the correlations of the 5-HT turnover involving the frontal cortices OFC, whereas the ratio in the mSN established negative correlations.

##### Correlative Analysis of NA Tissue Contents

We observed very few correlations between the NA content (17, comprising 9 positive and 8 negative correlations), keeping in mind that the correlations were conducted on a limited number of regions compared to the other parameters (data not shown). The FPN treatment decreased the number of correlations for the NA content (7, comprising 3 positive and 4 negative correlations). In the control group rats, the NA content in the NAc shell correlated with that in the IL and DRN. This pattern was completely lost in the FPN-treated rats. Likewise, the correlations of the NA content involving the hippocampus (dHP, vHP) and hypothalamus (dHY, vHY) were absent in the FPN-treated rats (data not shown).

##### Correlative Analysis between Neurotransmitter and Their Metabolites Tissue Contents in a Single Brain Region

**Figure 5:**
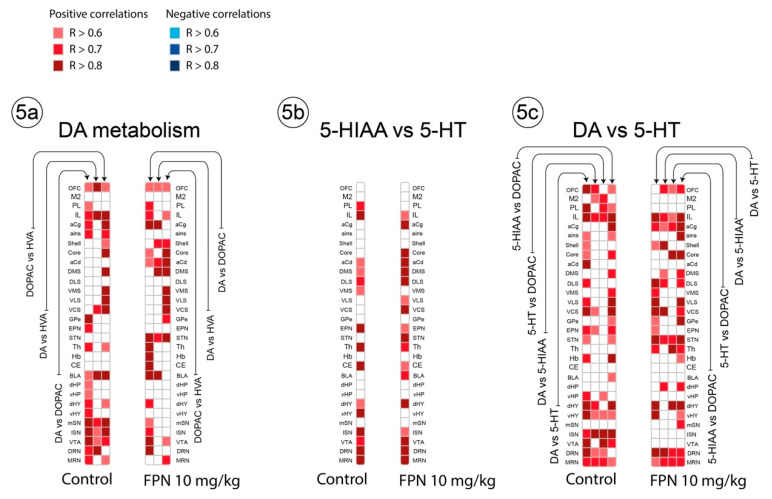
Correlative analysis between the DA and 5-HT neurochemical indices in a single brain region of rats. Representation of the range of Pearson’s R values for each linear regression within the DA system (**a**), 5-HT and 5-HIAA (**b**), DA and 5-HT systems (**c**), tissue contents (pg/mg) between the 30 brain areas in the control water-treated rats (first column) and the FPN 10 mg/kg-treated rats (second column). Colored boxes correspond to the existence of a correlation between the two parameters (all significant correlations are positive) considered after correction for multiple comparisons.

We studied the correlative link between the neurotransmitter and its metabolite(s) or between the metabolites themselves in single brain regions. The DOPAC content correlated with the DA or HVA contents in 18/30 and 17 brain areas of the control rats, respectively (Figure 5a). The HVA and DA content correlated less. Although FPN slightly decreased the number of correlations, the profile was not dramatically modified compared to the control group, and were still all positive.

The study of the correlations between 5-HT and its metabolite 5-HIAA was surprising (Figure 5b). The correlations reached significance in 15/30 and 18/30 regions in the water- and FPN-treated rats, respectively. The pattern of correlations was slightly different between the water- and FPN-treated rats, with stronger relationships in some striatal quadrants of the FPN-treated rats (Figure 5b).

We expanded the analysis of the correlations in a single brain region between the DA and 5-HT indexes, including DA and 5-HT, DA and 5-HIAA, 5-HT and DOPAC, and DOPAC and 5-HIAA (Figure 5c). We found that the number of correlations for all the pairs of compounds was elevated, and almost similar in the FPN-treated rats, particularly between 5-HIAA and DOPAC (19/30 regions in each group). The main differences are found at the level of the VTA, lSN, PL, and aCg (Figure 5c).

#### 2.3.2. Between Monoaminergic Systems

##### Correlative Analysis between the DA and 5-HT Neurochemical Indexes

**Figure 6:**
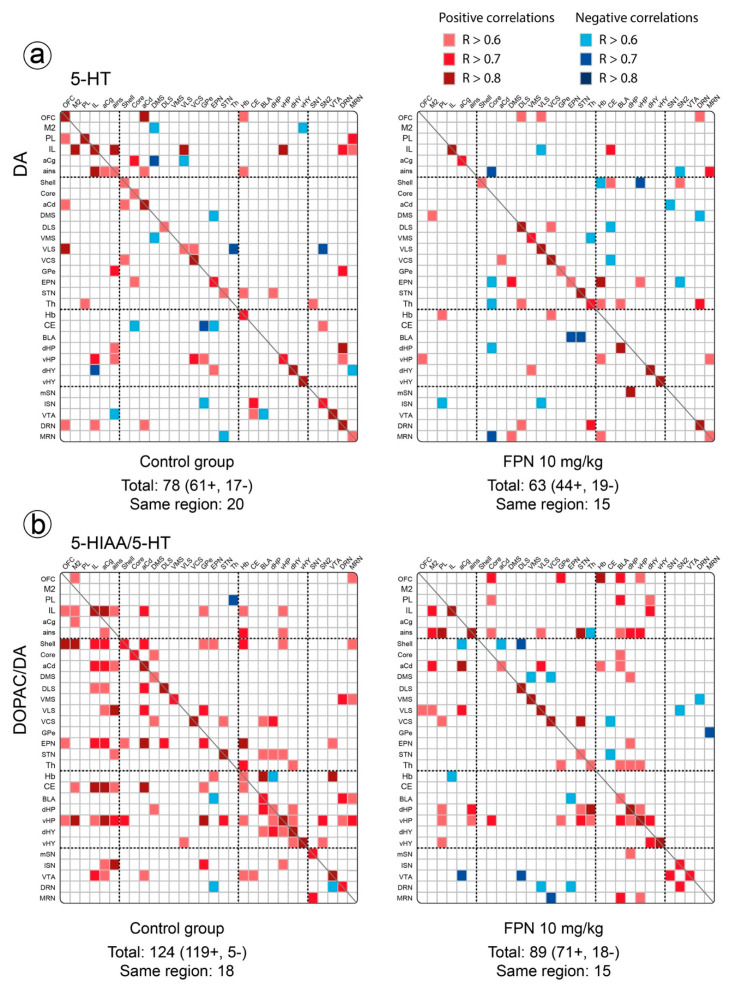
Correlative analysis between the DA and 5-HT neurochemical indices across the rat brain regions. Representation of the range of Pearson’s R values for each linear regression between the DA and 5-HT tissue contents (pg/mg) (**a**) and the DOPAC/DA and 5-HIAA/5-HT ratios (**b**) between the 30 brain areas in the control water-treated rats (first column) and the FPN 10 mg/kg-treated rats (second column). Colored boxes correspond to the existence of a correlation between the two parameters (red: positive; blue: negative) considered after correction for multiple comparisons.

The DA and 5-HT tissue contents displayed 78 correlations, including 19 in the same brain area (all positive) in the control rats (Figure 6a; see also Figure 5c). The regions establishing the most correlations were IL (10), ains (8), and VLS and vHP (7 each). The number of correlations decreased after the FPN treatment, notably in the correlations involving the cortical 5-HT and DA content within the cortex and toward the subcortical areas. Conversely, there was an increase in the correlations involving DA in the basal ganglia and 5-HT in the limbic regions (Figure 6a).

We observed a large number of correlations between the DOPAC/DA and 5-HIAA/5-HT ratios in both groups (124 correlations; Figure 6b). The number of correlations was higher for the vHP and NAc shell (22 and 17, respectively), and moderately high for the aCg, aCd, ains, EPN, CE, and Hb (9–12). The FPN treatment decreased the number of correlations between the DOPAC/DA and 5-HIAA/5-HT ratios. Both ratios correlated less within the basal ganglia, with a distinct pattern, and in the limbic regions. Conversely, there was a noticeable increase in the correlations established by the cortical DOPAC/DA ratio with the 5-HIAA/5-HT ratio in the basal ganglia or the limbic regions.

##### Correlative Analysis between NA and DA or 5-HT Tissue Contents

A total of 56 correlations were observed between the NA and DA contents, including 10 in the same brain area (all positive). The NA content in the cortex correlated less with DA in the limbic and mesencephalon regions in the FPN-treated rats. Similarly, the cortical DA correlated less with NA in the limbic and mesencephalic regions in the FPN-treated rats. The correlations of the NA content in the DRN with the DA content of eight different brain regions were reduced in the FPN-treated rats (data not shown).

The NA and 5-HT tissue contents displayed 39 correlations (comprising 28 positive and 11 negative correlations), including 13 in the same brain area (all positive) of the control group. The brain regions in which a higher number of correlations was found for the NA and 5-HT contents were the PL, ains, and vHP (6–7) and the aCg, the medial and lateral SN, and the MRN (5 each). The number of correlations slightly decreased after the FPN treatment (34, comprising 24 positive and 11 negative), including nine in the same brain area. There was no correlation of NA content in the mesencephalon with the 5-HT in the mesencephalon or limbic regions, as compared with the control water-treated rats. There was an increase in the correlations of the NA and 5-HT content in the basal ganglia of the FPN-treated rats (data not shown).

## 3. Discussion

In this study, we investigated the effect of chronic exposure to FPN (10 mg/kg/day for 21 days) on the monoamine tissue contents in 30 distinct regions of the rat brain. The FPN administration caused a marked deficit in the DA and 5-HT tissue contents in some brain regions, particularly the striatum. The qualitative analysis using correlations between pairs of brain regions revealed that FPN dramatically modified the pattern and balance of monoamines between the brain regions. The pattern of effect induced by FPN on the biogenic amines could correspond to a decrease in the cell energy and a destruction of the DA neurons.

We showed that the chronic administration of FPN significantly reduced the DA levels in a region-dependent manner and more particularly along the mesoaccumbal and mesostriatal tracts. The alterations were heterogeneously distributed at the level of the cell bodies and terminals. It also altered the 5-HT tissue content. Previous studies showed that FPN dose-dependently changed the activity of various enzymes involved in oxidative stress and inflammation in the brain when chronically administered at the 5–30 mg/kg range of doses [8,9,11,12,38]. Thus, the dose of 10 mg/kg, considered as a moderate dose, profoundly alters the neurochemistry of monoamines, particularly DA and 5-HT.

Our results extend previous studies reporting a decrease in striatal DA and TH-immuno-labelling upon the intra-nigral administration of FPN [14]. The inflammatory responses caused by the intra-nigral injection of FPN were revealed by the increased levels of proinflammatory factors, such as inducible nitric oxide synthase, cyclooxygenase 2, and tumor necrosis factor alpha in the SN and in the striatum, as well as by the up-regulation of glial fibrillary acidic protein expression and by the activation of microglia, demonstrated in turn by an increase in the ionized calcium-binding adaptor molecule 1 immunoreactivity in the striatum and SN. The increase in proinflammatory mediators in the FPN-treated rats was inversely correlated with the loss of nigrostriatal DA neurons, shown by a decrease in the TH immunoreactivity in the striatum and SN [14]. We have also reported, after the intra-nigral administration of FPN (15 and 25 µg), an about 50% decrease in the striatal DA tissue levels and a loss of nigral TH immune-labelling compared to the vehicle-treated rats [15]. The DA alterations were similar between the two intra-nigral doses of FPN, suggesting that the toxicity toward DA neurons had already reached its maximum at the lower dose administered. These data strongly suggest that the decrease in DA tissue content and its metabolites we report after chronic oral gavage includes neurodegeneration of some DA neurons, possibly related in part to an effect at the level of the SN.

A marked heterogeneity of the DA alteration was reported from the shell of the NAc to the ventro-caudal part of the striatum (VCS), paralleled by the heterogeneity reported at the level of regions housing DA cell bodies. Indeed, FPN had a low, and not significant impact, on the DA and its metabolite content in the VTA and the NAc shell, which is exclusively innervated by VTA DA neurons [31,32,39]. FPN had also lower noxious effects, notably for the DA metabolites, in the lateral SN, the ventrolateral part of the striatum (VLS), and the VCS, with the DA innervation of these latter striatal territories originating mainly from the lateral SN [32]. The main noxious effects are found at the level of the medial SN and its corresponding innervated striatal territories, including the anterior, the mediolateral part of the striatum, and part of the NAc core. The heterogeneity of the effects, at least with respect to the mesoaccumbens versus mesostriatal territories, has been previously reported with other toxins, notably MPTP [17]. Yet, it might not be only due to the specific and deleterious effect of FPN on the DA neurons of the medial SN. In fact, using the DOPAC/DA ratio, an indirect index of the DA turnover [40], we found that the turnover was increased more specifically in the VTA, the lateral SN, the VCS, and the VLS, exactly corresponding to the territories that were less affected by FPN for DA. These data indicate that the biochemical activity of DA neurons in the VTA, lateral SN, and VLS is enhanced. It is tempting to speculate that these territories are engaged in a neurodegenerative process as well, though delayed with respect to the medial SN. Factors other than a direct, poisoning action of FPN on medial SN DA neurons are likely involved in this intriguing pattern of defection, possibly degeneration. This idea is also supported by the fact that the DA content in other brain regions is poorly altered by FPN, and even increased.

Serotonin could be one of these external factors involved in the outcome of DA neurons. A decrease in the 5-HT concentrations is observed in some brain regions and notably in the brain regions where DA is altered. However, the 5-HIAA/5-HT ratio is largely enhanced in these brain regions. It could suggest the activity of 5-HT neurons is increased. This is an important parameter, because it is known that 5-HT can enhance the activity of DA neurons in particular when DA neurons are activated [24,41,42]. Such an excitatory effect has been well illustrated in the mechanism of action of the toxin MDMA and also with haloperidol and amphetamine [24]. It basically involves the stimulation of the 5-HT_2A_ receptor subtype [43,44,45,46,47], sustaining the enhanced synthesis of DA in the striatum and less in the NAc. Other state-dependent, excitatory influences involving 5-HT_3_ and 5-HT_4_ receptors have been also reported, principally acting at the level of DA cell bodies [48,49,50,51,52,53], but that can be observed in the striatum and the NAc in the case of the co-activation of DA and 5-HT release [48]. While we can postulate that the metabolic activity of 5-HT neurons is enhanced in some brain regions, we cannot really affirm that the decrease in the 5-HT tissue content reported in the striatum or the SN corresponds to a loss of 5-HT terminals in these regions. In fact, this loss of 5-HT content is poorly associated with corresponding changes in 5-HIAA, which can itself be enhanced in specific brain regions, such as the DMS. In addition, we have no evidence that the number of 5-HT cell bodies could be decreased by FPN. Rather, there is a tendency towards an increase in both the 5-HT and 5-HIAA content in the DRN and the MRN, and a significant increase in the 5-HIAA/5-HT ratio in both regions. It is noteworthy that the rise of the cytosolic concentrations of 5-HT and the increase in metabolism has been proposed for the destruction of 5-HT neurons induced by rotenone in vitro [54]. Additionally, the exposure of rats as neonates or adults or PC12 cells to the organophosphate chlorpyrifos or other neurotoxins enhanced the tryptophan hydroxylase expression and increased the 5-HT metabolism [55,56,57]. Thus, FPN is also a drug altering the function of 5-HT neurons. However, despite the existence of some anatomical specificities concerning the location of the 5-HT cell bodies innervating the brain [33,58], the neuronal origin of 5-HT cell bodies innervating the striatum is quite similar, thereby merely accounting for the heterogeneity of the 5-HT responses to FPN reported in adjacent striatal territories.

In order to study the possible defect of monoamine metabolism in the selected brain regions, we have addressed the relationship between a neurotransmitter and its metabolite(s) in single brain regions using a correlative analysis [29,37]. The relationships between the substrate and its products are intuitive in a single brain region, but the correlations are not always significant. This is likely due to the complexity of the metabolism of monoamines, which involves several enzymes, transporters, and cell partners. It is interesting to note that the number of correlations between DA and HVA (three enzymatic steps and different cell populations) was lower compared to DOPAC versus DA (two enzymatic steps, different cell populations) or DOPAC versus HVA (one enzymatic step, different cell populations) [28,36,40,59]. Moreover, the metabolism of monoamines differs between brain regions [30,40]. FPN slightly modified the pattern of correlations linking the substrate and its product for DA and 5-HT systems. The correlations were stronger in the striatal regions for both systems, whereas they were less pronounced for the DA system in mesencephalic regions. A last point to consider in the metabolism of monoamines is that they share some enzymes involved in their catabolism and notably monoamine oxidase A (MAO-A), at least in rodents [60,61]. As previously reported [30], there were several regions in which DA and 5-HT contents correlated. This could be related to the known interactions between the two systems, as briefly evoked above, and this could be also related to the shared catabolism. The finding that the DOPAC and 5-HIAA contents also correlated in several brain regions suggests that the 5-HT and DA metabolism are intermingled. Yet, this point is difficult to catch without specific approaches. It has been reported that the knock-out of MAO-A or the chronic blockade of MAO-A in mice produced an increase in the striatal 5-HT in the terminals of DA neurons [62,63]. Conversely DA can enter 5-HT terminals at least in the striatum [64]. Thus, the use of correlations presents the fair advantage to demonstrate that FPN slightly changed the pattern of correlations between DA and 5-HT-related compounds, particularly in the lateral SN. However, the pattern is not clearly paralleling the quantitative loss of DA and 5-HT in the striatum. Other modifications in the pattern of product/substrates correlations by FPN were found in the cortical regions, the STN, or the thalamus (Figure 5c), suggesting that the regional and quantitative loss of DA and 5-HT contents could be linked to broader system interactions.

In order to study possible changes in the system interactions, we studied the correlations for one or two neurochemical parameters between brain regions. This approach allows for qualitatively studying the balance of monoaminergic transmissions and their interplay or connectivity for a single monoaminergic system or between monoaminergic systems across multiple neurobiological networks [30]. In line with previous studies [29,30], the number of correlations for a single neurotransmitter system between pairs of brain regions was quite low for monoamines and their metabolite(s) and higher for the ratios in vehicle-treated rats. Several reasons could be evoked to account for the lack of consistency of these patterns in controls, including the species, the age, the handling or training in distinct behavioral experiments, the number of animals/per group, the conditioning of the samples, the chromatographic analysis, and the size and location of the punch [29,37,65]. Regarding the latter aspect, the size of the STN sampled was too large compared to previous punching procedures. The “STN” in our experiment represented only part of the tissue sampled, which surely leads to the modification of both quantitative and qualitative assessments. In any case, there are some interesting patterns that could be extremely pertinent for the understanding of the actions of FPN in the brain. While the profile of correlations obtained on DA is not drastically modified by FPN, the one obtained on DOPAC clearly shows that the correlations of DOPAC content between the striato-striatal regions, previously reported for the ratio DOPAC/DA [29], are almost suppressed in FPN-treated rats. Moreover, the correlations of the DOPAC/DA ratio between the cortical regions and cortico-basal ganglia regions are surprisingly low in the FPN-treated rats and, more generally, the number of correlations of the DOPAC/DA ratio in the whole brain was lower in the FPN-treated rats. We previously reported a lower number of correlations in the R6/1 mouse model of Huntington’s disease compared to the wild-type counterpart at a pre-symptomatic stage [37]. It preceded dramatic decreases in the striatal DA contents at later, symptomatic stages in those mice. Nonetheless, the reduction in the DOPAC/DA ratio does not constitute a signature of ongoing degenerative process, since we observed it with the 5-HT_2C_ receptor agonist lorcaserin [66], a drug known to oppose the behavioral consequences associated with an increase in DA transmission [67].

The data with FPN suggest that the coupling of DA transmissions between the cortical-subcortical regions is less efficacious. It is noteworthy that the profile of correlations between DA and 5-HT or between their respective ratios DOPAC/DA and 5-HIAA/5-HT across the brain similarly suggests the loss of the cortico-subcortical relationships. There was a major loss of correlations of the 5-HT content or the 5-HIAA/5-HT ratio of the mesencephalic regions toward other brain regions. When looking at the 5-HT/DA correlations or the correlations of their ratio in the same region, there is a decrease in the SN and an increase in the striatal quadrants. To further compare with previous examples found in the lab, the reduction in the DA correlations and/or the DOPAC/DA ratio in the brain induced by the 5-HT_2C_ receptor agonists WAY-163909 and lorcaserin occurred in the context of increased correlations for the 5-HT content and even for the NA content in the whole brain [65,68,69]. 5-HT and NA are the prototypical neuromodulatory systems of the brain, and their inability to correctly respond to the loss of DA connectivity can be part of a broader picture of a neurodegenerative process. This aspect could be studied with known neurotoxins altering DA neurons integrity, including 6-OHDA, MPTP, or rotenone. Thus, the pattern of DA and 5-HT quantitative defects is particular with FPN and could promote altered balances of monoaminergic tones modulating the cortico–subcortical interactions.

The reported alterations on monoamines suggest that FPN could predispose to developing numerous neuropsychiatric disorders in considering the cortical-subcortical defects and imbalances of monoamines, which can be associated with the modification of decision-making and behavioral traits [70,71]. However, we do not have evidence that the changes induced by FPN on monoamines are irreversible, and our study was not associated with a fine analysis of behavioral performance. It is noteworthy that, upon intra-nigral administration, the animals did not exhibit classical signs of experimental parkinsonism, except for the loss of motor coordination and the higher pain sensitivity [15]. The pattern of striatal DA depletion we report after FPN, mostly medial, does not correspond to the pattern of destruction in Parkinson’s disease, mostly lateral [72]. However, DA neurons are altered, with profound changes in the correlative pattern of monoamine markers across the brain, which could make our study interesting for the prodromal, pre-symptomatic phase of Parkinson’s disease or Huntington’s disease.

## 4. Material and Methods

### 4.1. Animals

Adult male Sprague Dawley rats (*n* = 24 rats equally distributed in two groups: 12 in control water-treated group and 12 in FPN-treated group) (Janvier Labs, Paris, France), weighing 300–350 g at the beginning of the experiments, were used in the study. The animals were housed in a group, 2 per cage according to the calculation of the size of the cage (model 2154F, 482 × 267 × 210 mm; surface: 940 cm^2^, Techniplast, France) and their weight, and were maintained under standard conditions with 12 h light/dark cycles (lights on at 8 am) at room temperature (22 ± 2 °C, 60% ± 5% humidity). They were fed with a standard pellet diet (A03 product, SAFE, Auxerre, France) and tap water ad libitum throughout the study. The rats were handled once daily in order to avoid the stress induced during handling and to familiarize them with the experimental male and female personnel. The treatment was performed between 09:00 and 16:00 h. All the procedures used were in accordance to European Economic Community (86-6091 EEC) and the French National Committee guidelines (décret 87/848, Ministère de l’Agriculture et de la Forêt) for the care and use of laboratory animals. The procedures were approved by the Ethical Committee of Centre National de la Recherche Scientifique, Région Aquitaine-Limousin, and the University (N°50120166-A, 17/01/2015).

### 4.2. Drug and Reagents

Fipronil (5-amino-1-[2,6-dichloro-4-(trifluoromethyl)phenyl]-4-[(trifluoromethyl)sulfinyl]- 1H-pyrazole-3-carbonitrile) (FPN) was provided by Sigma Aldrich, Dusseldorf Germany. All other reagents used were from available commercial sources.

### 4.3. Treatment

After handling for 10 days for acclimation to the vivarium conditions (UMR 5287 Institute, University of Bordeaux; agreement B33-063-269), the rats were divided in two treatment groups: the control group, which received drinking water (1 mL/kg); and the FPN-treated group, which received the pesticide (10 mg/kg) suspended in the same volume of drinking water as in the control group [38]. The rats were given chronic treatment once daily, by oral gavage, for 21 days. The weight of the rats was taken on daily basis.

The regimen of 10 mg/kg FPN upon oral administration for 3 weeks was chosen to ensure a steady state of its concentration and its metabolites [10]. Briefly, FPN (5–10 mg/kg oral gavage after single administration or two weeks administration) is rapidly metabolized in rats and mice to Fipronil sulfone [10,73] and gives, after the two weeks of treatment, an elevated concentration of hydroxy-fipronil in urine [73]. Indeed, the urine concentration of hydroxyl-fipronil reached 20 ng/mL after a single administration of 10 mg/kg, and above 10,000 ng/mL after 21 days of treatment of the same dose [73]. The half-life of fipronil sulfone was found to be 14 ± 3 days in plasma, 17 ± 2 days in the brain, and 26 ± 3 days in adipose tissue in mice after a single administration of 10 mg/kg. After chronic administration (10 mg/kg for three weeks), the concentrations in the brain, adipose tissue, and plasma of fipronil sulfone reached a steady state after 5 days of treatment until the end of the treatment [10]. The adipose tissue displayed the highest concentration of fipronil sulfone when compared to the brain and plasma.

### 4.4. Tissue Collection of Brain Regions

The rats were brought to the experimental room in their home cage 48 h after the last oral gavage. After a habituation period lasting at least two hours, the rats were sacrificed by decapitation using a guillotine in a quiet room next door. No anesthetics were used, because anesthetics quickly act on the metabolism of neurotransmitters and the brain connectivity [74], two parameters that we are reporting in this study. In addition, the efficacy to reach deep anesthesia differs between individuals, and the contribution of the effect of the anesthetic in our inter-individual analyses would have been impossible to determine. The brain was rapidly removed, immediately immersed in isopentane maintained at −35 °C for 3 min, and then stored in a deep freezer at −80 °C until use.

On the day of dissection, the rat brain was placed in a cryostat (Leica CM3000, Leica Biosystems, France) maintained at −24 °C and the brain areas were collected with the help of a rat brain atlas [75]. Using a magnifying glass, the discrete regions were identified and directly taken out from the brain using stainless steel cannulae of 500 or 800 µm inner diameter. The estimation of the depth of the taken tissue (usually ranging from 300 to 500 µm) is based on the number of rolls (60 µm each) to reach a plane surface, except for the STN. The STN was taken with the smaller cannula used as a spoon to collect the surface (usually around 200 μm thickness) of the tissue from the medial to the lateral extension of the STN. The operation was performed three to four times to account for the rostro-caudal length of the STN. The left and right parts of one discrete brain region were taken and pooled together in labelled and pre-weighed small Eppendorf tubes (0.6 mL volume). All the tissue samples were stored in a deep freezer at −80 °C until analysis.

The bilateral punches were taken from 30 distinct brain regions, including the motor cortex (M2); orbitofrontal cortex (OFC); prelimbic cortex (PL); infralimbic cortex (IL); anterior cingulate cortex (aCg); anterior insular cortex (ains); nucleus accumbens (NAc) shell and core; six parts of the striatum, including the anterior striatum (aCd), dorsomedial striatum (DMS), dorsolateral striatum (DLS), ventromedial striatum (VMS), ventrolateral striatum (VLS), and ventro-caudal striatum (VCS); the globus pallidus pars externa (GPe); the entopeduncular nucleus (EPN); the central (CE) and basolateral (BLA) nuclei of the amygdala; the dorsal and ventral parts of the hippocampus (dHP and vHP); the thalamus (Th); the dorsal and ventral hypothalamus (dHY and vHY); the subthalamic nucleus (STN); the Habenula (Hb); the substantia nigra (medial SN (mSN) and lateral SN (lSN)); the ventral tegmental area (VTA); and the dorsal and median raphe nuclei (DRN and MRN, one punch only). A camera was used to capture pictures of punches of all the brain regions. The location of the punches has been reported in Figure 1.

### 4.5. Tissue Processing and Neurochemical Analysis

On the day of the neurochemical analysis, the Eppendorf tubes containing the brain tissue of one region were retrieved from the deep freezer and placed on ice. The tubes were quickly wiped and weighed on the same precision balance [29]. In all cases, the size for each structure was not significantly different between the groups. The brain tissues were homogenized in 100 µL of 0.1 N HClO_4_, sonicated, and centrifuged at 13,000 rpm for 30 min at 4 °C (Eppendorf 5424R, Fisher Scientific, Illkirch, France). Aliquots (10 µL) of the supernatants were directly injected into the HPLC system coupled with electrochemical detection (HPLC-ECD).

### 4.6. Chromatographic Analysis

The tissue concentrations of the monoamines NA, DA, and 5-HT and their metabolites were measured using the HPLC-ECD system, as previously reported [65,68]. The samples were kept on ice after the centrifugation (series of 8 or 10 samples at maximum). Supernatants (10 µL) were injected using a manual injector equipped with a 20 µL loop (Rheodyne 7725i, C.I.L.-Cluzeau, Sainte-Foy-La-Grande, France) into an Equisil ODS (C18) HPLC column (150 × 4.6 mm, 5 µm; C.I.L.-Cluzeau) preceded by a Brownlee–Newgard precolumn (RP-8, 15 × 3.2 mm, 7 µm; C.I.L.-Cluzeau). The composition of the mobile phase was as follows: 70 mM of NaH_2_PO_4_, 0.1 mM of disodium EDTA, 2-Octane-sulfonic acid (concentration approximately corresponding to 130 mg/L of the mobile phase and adjusted to obtain the best separation between the electrochemical reactive eluents) in deionized water (18 MΩ.cm^−2^) containing 7% methanol. The pH was adjusted at approximately 4 with orthophosphoric acid to get a good separation of the eluents in the chromatogram. The mobile phase was filtered using a 0.22 mm Millipore filter. The temperature of the column was maintained at 40 °C. The mobile phase was delivered at a 1.200 mL/min constant flow rate using an HPLC pump (LC20-AD, Shimadzu, France).

The monoamines eluted from the column at different retention times (approximately: NA, 2.5 min; DOPAC, 3.7 min; DA, 5 min; 5-HIAA, 6.4 min; HVA, 8.4 min; and 5-HT: 12.8 min; Supplementary File), which then entered the coulometric detection cell (Cell 5011, ESA, Paris, France) equipped with two electrodes. The potential of the two electrodes was fixed at +350 mV and −270 mV on the coulometric detector (Coulochem II, ESA, Paris, France). In return, the detector detects the electrons at the level of the electrodes, the current generated by a compound being directly proportional to its injected quantity (quantitative method) over the tested ranges of concentrations. The coulometric detector was connected to a computer via an interface (Ulyss, Azur system, Toulouse, France). The Azur system allows visualizing the elution time and the amplitude of the different neurotransmitters and their metabolites.

Calibration curves were performed using a range of concentrations of eluents compatible with the expected quantities (ng range for DA in the striatum; pg range for the hippocampus). The changes of gain programmed during the acquisition were precisely set to increase the sensitivity of detection [65,68]. Standard solutions containing all the compounds of interest at known concentrations were systematically injected each day before and after a series of samples.

### 4.7. Statistical Data Analysis

The tissue levels of monoamines (NA, DA, and 5-HT) and their respective metabolites were expressed in pg/mg of tissue. The indirect index of the turnover corresponding to the ratio between the metabolite and its parent neurotransmitter (DOPAC/DA and 5-HIAA/5-HT) was also calculated. For each brain region, the data are presented as the mean ± SEM of values. Aberrant data were discarded on the basis of the value outside the range of the average mean ± two standard deviations [30].

The statistical analysis of the body weight was performed with a two-way repeated measures ANOVA (time × treatment). The statistical analysis of the neurochemical data was performed by using an unpaired Student’s *t*-test, comparing the data obtained in the control and FPN-treated rats for all eluents or ratios. The normality of the distribution was verified using the Shapiro–Wilk test for each parameter and ratio. In all cases, the criterion for significance was set at *p* < 0.05. Correlations were performed using Bravais–Pearson’s r correlation test for the content of each monoamine (*n* = 12/group before outliers; after outliers, *n* = 11/group for all brain regions, except for the Hb and the vHY, where we had *n* = 10/group). For each kind of multiple comparison analysis [30], within and between the monoamine systems, the p-values were adjusted using False Discovery Rate (FDR) controlling procedures [76]. The correlation was considered significant at the 5% level.

## 5. Conclusions

Our study shows that the chronic oral administration of FPN modified the DA and 5-HT levels in a few brain regions while sparing the NA levels to some extent. Thus, it confirms that FPN quantitatively induces marked alterations in nigrostriatal DAergic systems and extends these alterations to the raphe-striatal and raphe-nigral 5-HTergic systems, which appear to play an important role. In terms of environmental pollution, the dose used was likely high with respect to its concentration that could be absorbed [10,38], and it would deserve additional expertise at 3–10 times lower doses. Nonetheless, FPN or one of the studied metabolite - fipronil sulfone has a long half-life and can stay in the tissue for days [10]. In addition, the noxious effects of environmental toxins are rarely isolated, and the deleterious effects are presumably always linked to a combination of several factors, some being endogenous (stress), most of them being exogenous (heavy metals, pesticides). Unfortunately, its molecular targets are still unknown and its toxic effects, possibly related to some of its metabolites and their action on oxidative metabolism [73], would not be related to its primary site of action, namely the GABA receptors [77]. FPN should be considered with additional care by public health systems.

## 6. Research Highlights

- The effect of the chronic administration of fipronil on the monoamine tissue content is analyzed across 30 brain regions of rats.
- Fipronil non-homogeneously reduces dopamine and serotonin levels, the main defects being found in the striatum.
- Fipronil did not alter the noradrenaline tissue content, except in the nucleus accumbens.
- Fipronil completely modified the pattern of correlations, more prominently the dopamine and the serotonin turnovers, within and between brain regions.

## Figures and Tables

**Table 1:** (**a**). Tissue content of dopamine (DA) and its metabolites (pg/mg) in various brain regions of the control water- and FPN-treated rats; (**b**). Tissue content of noradrenaline (NA), serotonin (5-HT), and its metabolite 5-HIAA (pg/mg) in various brain regions of water- and FPN-treated rats.

(**a**)								
	**DA**		**DOPAC**		**HVA**		**DOPAC/DA**	
**Brain Region**	**Control**	**FPN**	**Control**	**FPN**	**Control**	**FPN**	**Control**	**FPN**
**OFC**	16.62 ± 3.99	12.93 ± 2.17	38.96 ± 4.65	37.54 ± 3.43	19.16 ± 2.89	15.84 ± 2.15	2.59 ± 0.37	3.09 ± 0.29
**M2**	28.20 ± 4.60	13.19 ± 0.98 *	57.28 ± 9.03	32.24 ± 3.01 *	16.13 ± 2.33	11.58 ± 2.22	2.39 ± 0.15	2.18 ± 0.22
**PL**	20.37 ± 2.71	35.56 ± 3 ***	66.65 ± 6.49	85.35 ± 7.00	28.42 ± 3.45	28.54 ± 2.73	3.66 ± 0.25	2.73 ± 0.25 *
**IL**	34.42 ± 8.14	29.02 ± 3.64	83.49 ± 6.36	109.7 ± 16.8	31.93 ± 5.25	36.85 ± 5.56	3.30 ± 0.33	3.65 ± 0.39
**aCg**	16.74 ± 2.15	16.62 ± 2.29	63.14 ± 8.34	75.58 ± 15.9	49.62 ± 5.96	60.72 ± 13.0	3.64 ± 0.26	4.24 ± 0.52
**ains**	38.46 ± 6.14	33.44 ± 4.75	77.47 ± 8.51	92.29 ± 13.59	81.96 ± 7.18	92.48 ± 10.3	2.05 ± 0.23	3.11 ± 0.55
**Shell**	1096 ± 92.6	845.9 ± 86	1643 ± 157	1581 ± 103	262.6 ± 17.9	286.6 ± 24.9	1.75 ± 0.26	1.85 ± 0.15
**Core**	1291 ± 80.3	941.0 ± 117 *	1601 ± 121	1194 ± 159	325.5 ± 26.4	260.5 ± 28.1	1.33 ± 0.08	1.45 ± 0.13
**aCd**	5039 ± 435	2432 ± 317 ***	2829 ± 128	1832 ± 186 ***	696.9 ± 44.33	435.9 ± 40.16 ***	0.58 ± 0.07	0.74 ± 0.09
**DMS**	2816 ± 220	1643 ± 129 ***	1523 ± 134	874.4 ± 71.6 ***	291.1 ± 20.4	178.9± 13.1 ***	0.53 ± 0.05	0.52 ± 0.03
**DLS**	2820 ± 206	1230 ± 160 ***	1159 ± 96.4	729.8± 63.5 ***	370.1 ± 22.5	261.2 ± 18.2 **	0.42 ± 0.04	0.63 ± 0.08 *
**VMS**	2835 ± 164	1124 ± 147 ***	1921 ± 199	1242 ± 120 **	432.9 ± 43.6	277.2 ± 24.5 *	0.79 ± 0.09	1.19 ± 0.17
**VLS**	2057 ± 274	1207 ± 162 *	1135 ± 127	1082 ± 113	357.3 ± 25.6	412.1 ± 37.5	0.54 ± 0.05	0.94 ± 0.15 *
**VCS**	1799 ± 139	1276 ± 170 *	689.6 ± 90.3	651.7 ± 65.9	199.7 ± 20.3	174.9 ± 15.0	0.38 ± 0.04	0.52 ± 0.05 *
**GPe**	66.24 ± 9.38	69.35 ± 11.1	101.9 ± 11.2	101.4 ± 10.8	64.18 ± 6.08	71.45 ± 9.26	1.51 ± 0.13	1.45 ± 0.14
**EPN**	88.01 ± 9.03	68.08 ± 8.54	58.89 ± 7.73	47.93 ± 8.55	16.44 ± 2.05	16.78 ± 3.69	0.62 ± 0.05	0.59 ± 0.05
**STN**	100.4 ± 7.34	92.42 ± 13.18	63.37 ± 4.43	65.97 ± 6.73	12.18 ± 1.08	12.48 ± 1.78	0.59 ± 0.05	0.59 ± 0.04
**Th**	8.39 ± 1.28	6.22 ± 1.01	13.20 ± 1.12	12.73 ± 1.30	4.43 ± 0.72	4.85 ± 0.75	1.75 ± 0.25	2.20 ± 0.19
**Hb**	16.19 ± 2.63	12.47 ± 1.53	23.45 ± 4.06	19.14 ± 3.86	7.76 ± 1.07	7.13 ± 1.08	1.41 ± 0.29	1.61 ± 0.16
**CE**	878.5 ± 90.6	645.7 ± 116	324.5 ± 22.2	244.9 ± 32.7	48.91 ± 3.81	51.22 ± 9.57	0.37 ± 0.04	0.40 ± 0.03
**BLA**	601.8 ± 99.3	575.1 ± 148	308.3 ± 35.6	435.3 ± 67.1	84.80 ± 8.90	97.84 ± 14.0	0.55 ± 0.06	0.81 ± 0.14
**dHP**	2.33 ± 0.50	2.06 ± 0.42	5.90 ± 1.01	9.34 ± 0.94 *	4.82 ± 0.79	4.94 ± 0.64	2.18 ± 0.33	4.88 ± 1.25
**vHP**	3.82 ± 1.49	2.87 ± 0.75	6.93 ± 0.45	8.56 ± 0.55 *	3.44 ± 0.33	4.96 ± 0.39 **	2.91 ± 0.65	3.34 ± 0.59
**dHY**	72.06 ± 9.62	81.76 ± 9.32	42.97 ± 6.17	44.79 ± 3.99	4.90 ± 0.95	5.01 ± 0.42	0.59 ± 0.05	0.55 ± 0.04
**vHY**	45.43 ± 7.24	33.79 ± 3.92	44.94 ± 5.48	43.97 ± 8.65	5.51 ± 0.49	6.85 ± 0.71	0.84 ± 0.09	1.23 ± 0.28
**mSN**	318.5 ± 52.2	157.6 ± 27.5 *	157.8 ± 16.1	93.94 ± 12.5 *	55.25 ± 5.41	28.68 ± 4.65 **	0.52 ± 0.04	0.62 ± 0.06
**lSN**	227.2 ± 33.5	139.3 ± 16.8 *	86.04 ± 13.3	64.97 ± 8	27.00 ± 4.07	22.18 ± 2.87	0.36 ± 0.02	0.48 ± 0.04 *
**VTA**	319.3 ± 58.8	291.8 ± 50.6	534.5 ± 97.5	689.3 ± 127	142.8 ± 13.1	124.9 ± 13.2	1.63 ± 0.09	2.28 ± 0.19 **
**DRN**	53.78 ± 8.23	51.09 ± 7.30	34.64 ± 2.93	30.31 ± 3.40	9.38 ± 1.29	10.35 ± 1.27	0.69 ± 0.07	0.61 ± 0.06
**MRN**	43.85 ± 4.64	34.31 ± 3.95	32.41 ± 2.63	27.29 ± 2.02	25.41 ± 2.53	22.53 ± 2.49	0.61 ± 0.05	0.79 ± 0.07 *
(**b**)								
	**NA**		**5-HT**		**5-HIAA**		**5-HIAA/5-HT**	
**Brain Region**	**Control**	**FPN**	**Control**	**FPN**	**Control**	**FPN**	**Control**	**FPN**
**OFC**	nd	nd	89.67 ± 15.79	73.27 ± 7.68	594.3 ± 65.1	560.4 ± 68.1	6.99 ± 0.86	7.62 ± 0.74
**M2**	nd	nd	170.5 ± 24.3	140.8 ± 19.2	675.5 ± 82.6	628.3 ± 46.8	4.14 ± 0.70	5.73 ± 0.75
**PL**	43.15 ± 6.33	36.07 ± 3.83	55.77 ± 7.74	49.76 ± 5.67	362.8 ±38.4	404.9 ± 32.2	6.60 ± 0.51	8.67 ± 1.04
**IL**	52.20 ± 10.4	53.94 ± 6.99	83.63 ± 16.8	67.84 ± 8.41	415.8 ± 50.6	516.9 ± 73.1	5.16 ± 0.67	7.50 ± 0.88 *
**aCg**	66.20 ± 5.14	69.99 ± 8.57	45.75 ± 4.69	44.06 ± 6.67	348.3 ± 43.3	543.4 ± 80.0 *	7.87 ± 1.09	10.34 ± 0.94
**ains**	98.14 ± 14.4	93.90 ± 7.73	174.4 ± 19.5	184.3 ± 16.7	645.9 ± 40.9	629.2 ± 68.4	3.79 ± 0.45	4.09 ± 0.53
**Shell**	120.4 ± 15.3	115.3 ± 15.4	136.1 ± 16.0	89.27 ± 10.4 *	300.4 ± 20.78	310.1 ± 24.57	2.25 ± 0.29	3.62 ± 0.49 *
**Core**	75.38 ± 4.91	50.29 ± 7.38 *	114.3 ± 5.98	80.13 ± 6.81 **	276.6 ± 21.2	244.7 ± 36.4	2.58 ± 0.17	2.91 ± 0.22
**aCd**	nd	nd	97.66 ± 7.04	70.03 ± 9.71 *	410.2 ± 18.5	347.4 ± 32.8	4.12 ± 0.21	5.21 ± 0.42 *
**DMS**	nd	nd	59.45 ± 4.89	57.12 ± 8.68	199.2 ± 16.8	405.57± 46.4 ***	3.18 ± 0.23	7.03 ± 0.45 ***
**DLS**	nd	nd	68.68 ± 6.04	56.19 ± 9.29	264.7 ± 24.0	244.6 ± 28.7	3.57 ± 0.17	4.47 ± 0.33 *
**VMS**	nd	nd	199.8 ± 17.4	115.9 ± 15.0 *	459.1 ± 50.5	352.0 ± 30.4	2.83 ± 0.15	3.08 ± 0.32
**VLS**	nd	nd	67.55 ± 8.54	42.08 ± 4.76 *	242.0 ± 19.1	227.6 ± 28.6	3.56 ± 0.28	5.58 ± 0.44 ***
**VCS**	49.96 ± 3.77	37.16 ± 5.16	184.5 ± 15.9	113.3 ± 18.2 **	235.3 ± 20.7	182.7 ± 21.2	1.27 ± 0.11	1.64 ± 0.09 *
**GPe**	nd	nd	181.7 ± 11.9	211.5 ± 29.7	666.7 ± 80.4	602.4 ± 64.3	3.40 ± 0.41	2.84 ± 0.37
**EPN**	95.32 ± 9.49	80.36 ± 13.05	219.1 ± 22.3	158.2 ± 22.7	627.9 ± 62.8	414.5 ± 43.4 *	2.71 ± 0.12	2.67 ± 0.20
**STN**	284.9 ± 18.72	266.6 ± 30.3	359.2 ± 30.5	267.1 ± 38.1	846.7 ± 62.9	741.2 ± 63.4	2.27 ± 0.19	2.43 ± 0.12
**Th**	185.5 ± 8.99	172.2 ± 15.3	100.0 ± 16.8	84.82 ± 7.43	530.9 ± 51.9	507.4 ± 41.2	5.78 ± 0.76	5.69 ± 0.56
**Hb**	53.9 ± 7.66	46.58 ± 6.13	122.8 ± 22.3	103.2 ± 17.5	390.7 ± 66.1	412.7 ± 50.1	3.31 ± 0.85	3.42 ± 0.55
**CE**	184.9 ± 20.9	184.4 ± 19.1	250.1 ± 32.6	201.7 ± 24.5	442.3 ± 63.2	382.8 ± 40.5	1.74 ± 0.16	1.93 ± 0.17
**BLA**	126.0 ± 9.29	99.37 ± 11.1	214.5 ± 18.1	181.3 ± 28.9	428.2 ± 27.6	451.3 ± 37.3	1.97 ± 0.21	2.72 ± 0.33
**dHP**	218.9 ± 21.1	223.5 ± 16.1	133.3 ± 15.9	121.3 ± 14.2	428.1 ± 46.8	480.6 ± 35.3	3.21 ± 0.40	4.19 ± 0.68
**vHP**	191.8 ± 22.5	231.1 ± 18.3	165.3 ± 19.9	187.9 ± 21.8	684.9 ± 50.7	830.4 ± 92.8	3.91 ± 0.51	5.23 ± 0.59
**dHY**	702.6 ± 143	645.9 ± 137	253.6 ± 34.4	219.2 ± 22.3	522.9 ± 71.5	509.0 ± 48.9	2.03 ± 0.14	2.28 ± 0.11
**vHY**	636.9 ± 111	510.5 ± 55.9	171.4 ± 19.2	143.5 ± 16.1	392.2 ± 43.7	503.6 ± 71.7	2.27 ± 0.19	3.46 ± 0.56
**mSN**	119.4 ± 13.4	95.23 ± 19.4	845.4 ± 46.6	501.4 ± 62.3 ***	941.5 ± 35.1	771.2 ± 72.4 *	1.14 ± 0.05	1.52 ± 0.17
**lSN**	121.3 ± 13.3	109.3 ± 9.69	500.1 ± 54.6	544.6 ± 52.5	574.1 ± 74.9	731.7 ± 54.1	1.11 ± 0.05	1.36 ± 0.11
**VTA**	474.1 ± 38.9	370.9 ± 47.2	688.2 ± 60.8	494.2 ± 79.0	885.5 ± 88.9	728.3 ± 86.1	1.24 ± 0.09	1.50 ± 0.09
**DRN**	568.9 ± 83.8	501.9 ± 70.9	1150 ± 151	1228 ± 193	2119 ± 186	2279 ± 317	1.65 ± 0.07	1.87 ± 0.06 *
**MRN**	352.4 ± 30.6	302.8 ± 38.4	1183 ± 101	1125 ± 121	2960 ± 262	3206 ± 370	2.47 ± 0.09	2.84 ± 0.07 **

Results are expressed as mean ± SEM values (pg/mg of tissue) in various brain regions of the control water- and FPN-treated rats, except for the DOPAC/DA ratio and the 5-HIAA/5-HT ratio. Starting from 12 rats per group, the final number of observations/group after the outliers is 11 for each parameter and brain region, except in vHY and Hb, where *n* = 10. * *p* < 0.05, ** *p* < 0.01, *** *p* < 0.001 (Student’s *t*-test). nd—not detected.

## Supplementary Materials

The following are available online at https://www.mdpi.com/1422-0067/21/16/5711/s1, Figure S1: Examples of chromatograms.

## Abbreviations

aCd	anterior caudate
aCg	anterior cingulate cortex
ains	anterior insular cortex
BLA	basolateral nucleus of amygdala
CE	central nucleus of amygdala
CNS	central nervous system
core	core of the nucleus accumbens
DA	dopamine
dHP	dorsal part of hippocampus
dHY	dorsal part of hypothalamus
DLS	dorsolateral striatum
DMS	dorsomedial striatum
DOPAC	3,4-dihydroxyphenylacetic acid
DRN	dorsal raphe nucleus
EPN	entopeduncular nucleus
FPN	fipronil
GABA	γ-aminobutyric acid
GABA_A_ receptor	γ-aminobutyric acid A receptor
GPe	the globus pallidus pars externa
Hb	habenula
HPLC	high pressure liquid chromatography
HVA	homovanillic acid
IL	infralimbic cortex
M2	motor cortex M2
MAO-B	monoamine oxidase-B
MDMA	3,4-methylenedioxy-methamphetamine
MPTP	1-methyl-4-phenyl-1,2,3,6-tetrahydropyridine
MRN	median raphe nucleus
NA	noradrenaline
NAc	nucleus accumbens
OFC	orbitofrontal cortex
PD	Parkinson’s disease
PL	prelimbic cortex
rpm	revolutions per minute
5-HIAA	5-hydroxyindoleacetic acid
5-HT	5-hydroxytryptamine; serotonin
shell	shell of the nucleus accumbens
SN	substantia nigra
lSN	lateral part of substantia nigra
mSN	medial part of substantia nigra
STN	subthalamic nucleus
Th	thalamus
TH	tyrosine hydroxylase
VCS	ventro-caudal striatum
vHP	ventral part of hippocampus
vHY	ventral part of hypothalamus
VLS	ventrolateral striatum
VMS	ventromedial striatum
VTA	ventral tegmental area
6-OHDA	6-hydroxydopamine
