# Supplementary material for: Chronic Administration of Fipronil Heterogeneously Alters the Neurochemistry of Monoaminergic Systems in the Rat Brain
Source: Int J Mol Sci. 2020 Aug 9;21(16):5711. doi: 10.3390/ijms21165711 (PMC7461054; doi:10.3390/ijms21165711)
Supplement: Supplementary file 1 [file ijms-21-05711-s001.pdf]

SUPPLEMENTARY FILE

Chronic administration of Fipronil heterogeneously alters the neurochemistry of monoaminergic systems in the rat brain

Rahul Bharatiya[1,2], Abdeslam Chagraoui[3,4], Salomé De Deurwaerdere[2], Antonio Argiolas[1,5,6], Maria Rosaria Melis[1,5], Fabrizio Sanna[1*], Philippe De Deurwaerdere[2*].

[1] Department of Biomedical Sciences, Section of Neuroscience and Clinical Pharmacology, University of Cagliari, SS 554, km 4,500, 09042 Monserrato, Cagliari, Italy; bhartiyarahul20@gmail.com (R.B.); argiolas@unica.it (A.A.); mrmelis@unica.it (M.R.M.); fabrizio.sanna@unica.it (F.S.)

[2] Centre National de la Recherche Scientifique (Unité Mixte de Recherche 5287), 146 rue Léo Saignat, B.P.281, F-33000 Bordeaux Cedex, France; salome.de-deurwaerdere@etu.u-bordeaux.fr (S.D.D.); deurwaer@u-bordeaux.fr (P.D.D.)

[3] Neuronal and Neuroendocrine Differentiation and Communication Laboratory, Institute for Research and Innovation in Biomedicine of Normandy (IRIB), Normandie Univ, UNIROUEN, INSERM, U1239, CHU Rouen, 76000 Rouen, France; abdeslam.chagraoui@univ-rouen.fr (A.C.)

[4] Department of Medical Biochemistry, Rouen University Hospital, 76000 Rouen, France.

[5] Centre of Excellence for the Neurobiology of Addictions, University of Cagliari, Cagliari, Italy.

[6] Institute of Neuroscience, National Research Council, Cagliari Section, University of Cagliari, Cagliari, Italy.

*These authors have equally participated.

Corresponding authors:

Dr Fabrizio Sanna, PhD: fabrizio.sanna@unica.it (F.S.); Tel.: +39-070-675-4330

Prof. Philippe De Deurwaerdere, PhD : deurwaer@u-bordeaux.fr (P.D.D.);

Tel.: +33-557-571-290

Running title: Chronic fipronil and brain monoamines

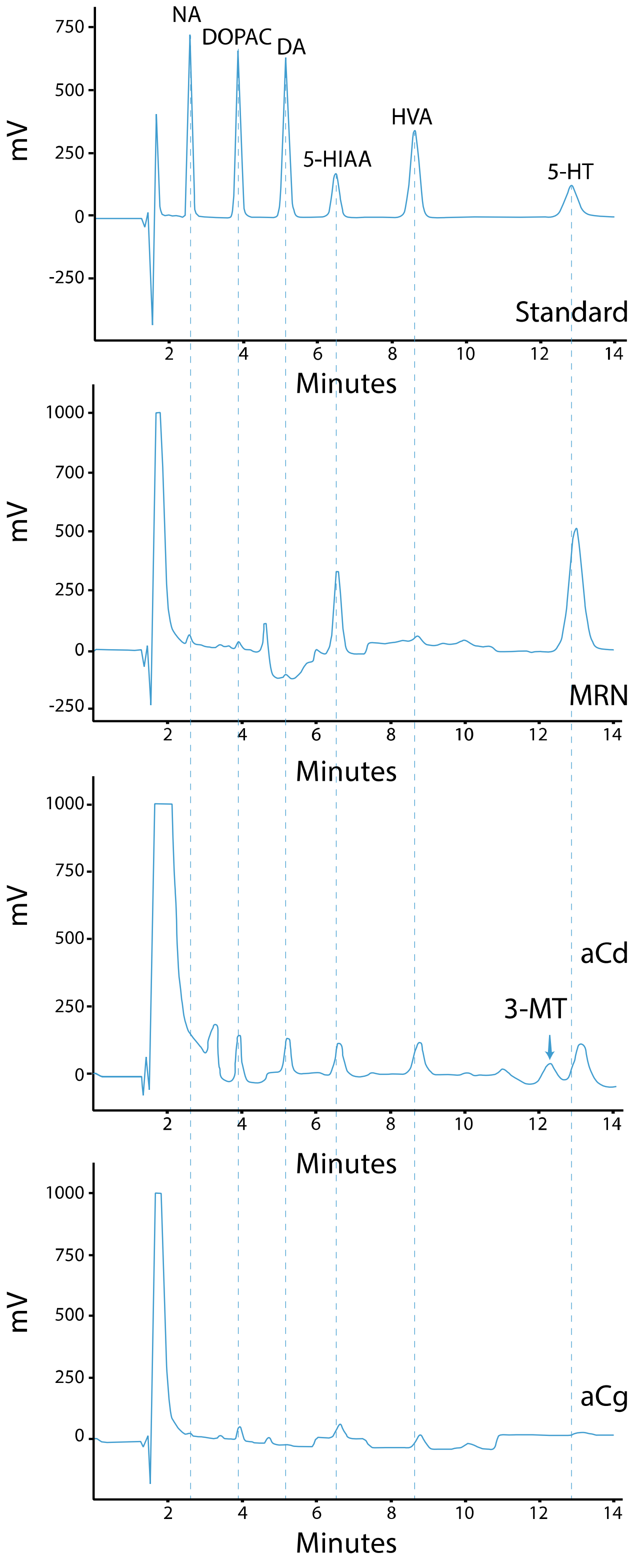

Figure S1: Examples of chromatograms

The purpose of these chromatograms is to illustrate the conditions of analysis and the heterogeneity of the chromatograms according to the brain regions analysed. The figure reports the electrochemical signals obtained for noradrenaline (NA), l-dihydroxyphenyl acetic acid (DOPAC), dopamine (DA), 5-hydroxyindoleacetic acid (5-HIAA), homovanillic acid (HVA), and serotonin (5-HT) in a standard solution (1 ng for each), the median raphe nucleus (MRN), the anterior caudate (aCd), and the anterior cingulate cortex (aCg). The gain was set at 200 nA for the standard solution. The gain was changing during the acquisition for each compound according to the analysed brain region as follows (in nA): DOPAC: 20, 200, 10 in MRN, aCd, aCg; DA: 20, 1000, 10 in MRN, aCd, aCg; 5-HIAA: 100, 20, 20 in MRN, aCd, aCg; HVA: 10, 100, 5 in MRN, aCd, aCg; 5-HT: 50, 20, 50 in MRN, aCd, aCg. The pic of NA was always acquired with a gain of 200 nA because of its proximity to the solvent front, reducing our ability to detect it in some brain regions despite the high sensitivity (see standard). It is the reason why the signal for NA was lost in some brain regions where the NA content is lower (see aCd). Note that the chromatographic conditions need additional expertise to determine the time of elution of other compounds such as 3-methoxytyramin (3-MT) which can be confounded with 5-HT in brain regions enriched with DA fibers such as the striatum. The example show that 3-MT is separated from 5-HT. In all cases, 10µl were injected into the HPLC system. The potential of the electrode was set at 350 mV (see materials and methods).
